# Collaboration for the inclusion of students with disabilities in education in Gondar, Ethiopia

**DOI:** 10.4102/ajod.v14i0.1569

**Published:** 2025-03-10

**Authors:** Mikyas Abera, Grace L. Francis, Ansha N. Ahmed, Solomon Dawud, Mohammedsani Ali, Gebrekidan Shibabaw, Bilen M. Araya, Shana J. Haines, Heather M. Aldersey

**Affiliations:** 1 Department of Sociology, College of Social Sciences and Humanities, University of Gondar, Gondar, Ethiopia; 2 Division of Special Education and Disability Research, College of Education and Human Development, George Mason University, Virginia, United States; 3 Department of Preventive Medicine, School of Public Health, Addis Ababa University, Addis Ababa, Ethiopia; 4 Department of Rehabilitation Science, School of Rehabilitation Therapy, Queen’s University, Kingston, Ontario, Canada; 5 Department of English Language and Literature, College of Social Sciences and Humanities, University of Gondar, Gondar, Ethiopia; 6 Department of Law, Faculty of Law, University of Gondar, Gondar, Ethiopia; 7 Department of Clinical Midwifery, College of Medicine and Health Sciences, University of Gondar, Gondar, Ethiopia; 8 Department of Education, College of Education and Social Services, University of Vermont, Vermont, United States

**Keywords:** students with disabilities, Gondar, Ethiopia, collaboration, inclusion, education

## Abstract

**Background:**

In Ethiopia, children with disabilities face significant barriers to education, which are exacerbated for those living in remote villages and areas affected by conflict and insecurity. Several studies have highlighted the important role that sustained multistakeholder collaborations could play in removing barriers to inclusive education, supporting students with disabilities and helping countries like Ethiopia achieve inclusion.

**Objectives:**

This study explored stakeholders’ strategies and the programmes and support they provide to schools or students with disabilities (K-12) to promote inclusive education in the central Gondar zone, Ethiopia.

**Method:**

This study used key informant interviews to collect detailed information on education stakeholders’ collaborations to support the inclusion of children with disabilities. The interviewees include experts, administrators and policymakers from purposively selected governmental and civil society organisations and schools.

**Results:**

This study found that collaboration focused on promoting accessibility, students enrolment and retention, financial, material and medical support, capacity-building, and institutional accountability.

**Conclusion:**

This study concluded that persistent instability and conflict hindered stakeholders’ collaborative efforts in the region. It also argued that structured or semi-structured collaborations are more effective for promoting inclusive education.

**Contribution:**

This article presents research findings on collaboration among educational stakeholders to promote inclusive schools and support students with disabilities. Its holistic approach identifies ecological and institutional factors that affect collaborations for inclusion, as well as the support and services that could be further explored in future research. Additionally, it highlights the lessons that education programmes could use to enhance community and stakeholder participation in school inclusivity.

## Introduction

Studies in low- and middle-income countries identified multiple and complex systemic, institutional and personal barriers to accessible and inclusive education for children with disabilities (Ginja & Chen 2021; Jones, Seager & Yadete et al. 2021). At a system level, children with disabilities face cultural values, attitudes towards people with disabilities and policy frameworks that do not support inclusive education (Mizunoya, Mitra & Yamasaki 2018). Widespread conflict and insecurity like the scenario prevailing in Ethiopia’s Amhara region disproportionately affect children with disabilities and their schooling (Bakhshi, Babulal & Trani 2018; Trani et al. 2014). In schools, these children often grapple with inaccessibility, a lack of resources and discriminatory and exclusionary practices (Genovesi et al. 2022). Their interpersonal relations suffer from undesirable peer pressure, stigma and stereotypes, affecting their educational performance and development (Chatzitheochari & Butler-Rees 2023; Shifrer 2013). Importantly, these barriers intersect, multiply, and compound their challenges in schooling, producing uneven educational outcomes and life chances for them.

All children, including children with disabilities, need education to grow, improve their status, make informed decisions and contribute to the development of inclusive and democratic societies (Hendricks 2007; Negash 2006; Wondimu 2004). International and national efforts have embraced these rationales and have promoted education for all, guided by the principles outlined in various key documents. These include the Declaration of Education for All (UNESCO 1990), the UN Convention on the Rights of the Child (UN 1990), the Salamanca World Conference on Special Needs Education (UNESCO 1994a, 1994b), the World Education Forum (UNESCO 2000), the Incheon Declaration (UNESCO 2015), Sustainable Development Goals (UNDP 2015) and the UNESCO Education 2030 Framework for Action (UNESCO 2015).

In Africa, additional frameworks have been established to advocate for the rights of all children, including those with disabilities, to receive quality and inclusive education. These frameworks include, primarily, the African Charter on the Rights and Welfare of the Child (OAU 1999) and the African Disability Protocol (AU 2018). Ethiopia recognises these instruments and has made significant strides in promoting inclusive education for children with disabilities. It has signed the Convention on the Rights of Persons with Disabilities (UN 2006), and its Constitution mandates the government to allocate resources to provide education and support for all children, including those with disabilities (GoE 1995). The Education and Training Policy (ETP) provides for supporting and building institutional and staff capacities for special needs and inclusive education (SNIE) (GoE 1994). Since 1997, Ethiopia has been implementing successive 5-year Education Sector Development Programmes, with special attention to disadvantaged groups, including children with disabilities (MoE 2010, 2021). The National Plan of Action on Disability (MoLSA 2012) and the Master Plan for Special Needs/Inclusive Education (MoE 2016) promote SNIE for children with disabilities – specifically, focussing on increasing enrolment at all levels and training (pre- and in-service) teachers on SNIE. Ethiopia also has additional and relevant frameworks for inclusive education, including the Strategy for SNIE (MoE 2012b), Guidelines for Curriculum Differentiation and Individual Educational Programmes (MoE 2012a), Guidelines for Establishing and Managing Inclusive Education Resource Centers (MoE 2015) and the Ethiopian Education Roadmap, 2017–2035 (Teferra et al. 2018). However, despite these policy commitments, many Ethiopian children with disabilities continue to face significant barriers to their school participation and performance (Ginja & Chen 2021; Jones et al. 2021; Temesgen 2018).

Meeting international and continental commitments that it has entered into and domesticated as laws, policies, programmes and guidelines to promote inclusive education requires Ethiopia to identify effective mechanisms to tackle the multilayered factors that obstruct the education of children with disabilities. In this respect, several authors underlined the key role of multisectoral collaboration in successful, sustained and intentional interventions to remove inclusion barriers for children with disabilities (Ainscow 2020; Hansen et al. 2020; Hunt et al. 2001; Tekola et al. 2023), especially for those in conflict situations (Kadir et al. 2018). They argue that collaboration enables several stakeholders to work together and achieve policy, programme or activity outcomes (Holland & Fitzgerald 2023; Salunke & Lal 2017; Williams 2015; Wood & Gray 1991). In inclusive education, specifically, potential and relevant stakeholders include local governments, education authorities, parents, schools, civil society and the private sector that create shared spaces to galvanise their expertise, resources, among others, to support inclusion (Ainscow 2020; Hansen et al. 2020; Hunt et al. 2001; Tekola et al. 2023). It is a complex process that involves intersecting individuals, institutions and systems (Ainscow & Sandill 2010; Rose & Doveston 2015), each with interests or agendas that may not necessarily align with those of others or the collaboration itself (Wood & Gray 1991). Its success depends on cultivating shared interests while embracing differing priorities whose pursuit should not prevent achieving the shared goals (Lax & Sebenius 1986; Wood & Gray 1991).

In this article, we explored the dynamic and complex process of multistakeholder collaboration for inclusive schools – along with its outcomes, challenges and prospects – in selected communities in Northwestern Ethiopia. It has been argued that ‘a multifaceted approach driven by various stakeholders’ is indispensable to, for instance, build awareness, provide training and mobilise resources towards inclusive education (Franck & Joshi 2017). In that spirit, a systems approach offers a holistic perspective in understanding multi-stakeholder educational collaboration in terms of interactions among participants and with their socioeconomic, cultural and political environment (Berger & Luckmann 1966; Luhmann 1995). It helps us identify the enabling or inhibiting effects of internal factors (such as collaboration) and external factors (such as the environment) including culture, discourses, policy, school structure, interactional practices and shared agendas on the capabilities of schools or collaborations to promote inclusion (Holland & Fitzgerald 2023; Rapp & Corral-Granados 2021).

This study adds to the growing literature on systemic exploration of multistakeholder collaboration in education (Hansen et al. 2020; Karimi-Aghdam 2017; Makoelle & Merwe 2014; Rapp & Corral-Granados 2021). Systems theory has also been used to study the collaborative approach as it applies to other sectors, including healthcare (McCovery & Matusitz 2014), development (Parmigiani & Rivera-Santos 2011), governance (Bardach & Lesser 1996; Getha-Taylor & Morse 2013) and community mobilisation and development (Huxham & Vangen 2000; Keast, Brown & Mandell 2007). In this study, we used systems theory to explore collaborations involving governmental organisations (GOs), civil society organisations (CSOs) and schools to support inclusion in schools. We conceptualised schools and collaborations as adaptive systems and explored the question ‘How do educational stakeholders collaborate to make schools more inclusive and accessible for students with disabilities in Gondar, Ethiopia?’ Using the systems theory, the study identified collaborative patterns that education stakeholders used to provide normative services and support the inclusion of children with disabilities in schools.

## Research methods and design

### Design and approach

This qualitative-descriptive study (Cohen, Manion & Morrison 2005; Sandelowski 2000) sought to understand multistakeholder collaboration to support the inclusion of K-12 students with disabilities in Gondar, Ethiopia. It gathered qualitative data through key informant interviews and iteratively coded and thematised our findings pertinent to the research question.

### Study sites

This study was conducted in the central Gondar zone of the Amhara region, Ethiopia. The zone is one of the 13 zones that make up the Amhara region and has its administrative capital in Gondar city.

For this study, four *Kebeles* [*parishes or neighbourhoods*] in four *Woredas* [*districts*] of the central Gondar zone were purposefully selected: *Kebele*-02 in Gondar city; *Kebele*-02 in Qola-Diba; *Kebele*-02 in Delgi and *Kebele*-01 in Chilga. *Kebele* is the lowest and smallest administrative unit in the Ethiopian federal governance structure. Since 1995, Ethiopia has had a federal governance structure of federal or national, region, zone, *Woreda* or sub-city and *Kebele* – in descending size and jurisdiction.

The first study site was *Kebele* 04 of Gondar City. *Kebele-04* hosts the Tsadiku Yohannes General Elementary School, where 203 students with disabilities attend special and inclusive classes that accommodate children with varied disabilities. The school has diverse community resources and support from GOs and CSOs (that include nongovernmental organisations [NGOs], Organisations of Persons with Disabilities [OPDs], Community-based Rehabilitation [CBR] and Community-based Organisations [CBOs]). The second study site was *Kebele-02* of Qola-Diba town, located 40 km from Gondar city. *Kebele-02* hosts the Qola-Diba General Elementary School, among others, which enrolled 54 students with disabilities. The school had relatively diverse community support for vulnerable groups, including the University of Gondar’s Community-based Rehabilitation (UoG-CBR) programme. The third study site was *Kebele-02* of Delgi town, located 90 km from Gondar city. Its comprehensive school enrolled 40 students with disabilities. The fourth and final study site was *Kebele-01* of Aykel town in Chilga *Woreda* (district), located 60 km from Gondar city. The town has nine educational institutions of different levels and has been significantly affected by persistent ethnic-based conflicts. The town availed a range of support to vulnerable children from GOs and CSOs.

### Sampling and recruitment

Based on maximum variation sampling (Patton 1990), study sites were selected to represent diverse settlements (urban, semi-urban and rural), socioeconomic statuses and community resources for inclusive education. Specifically, the study covered one urban site with low socioeconomic status residents (*Kebele-04*, Gondar city), two semi-urban sites (one with a higher [*Kebele-01*, Chilga] and another with a lower [*Kebele-02*, Qola-Diba] level of community resources – GO and CSO support) and one rural site (*Kebele-02*, Delgi) (see Table 1).

**TABLE 1:** Interviewees by type of organisation and study site.

Types of organisation	Study sites					*n*
	Qola-Diba	Chilga	Delgi	Gondar	Amhara Region	
Civil society organisation (CSO)	1	2	1	3	2	9
Governmental organisations (GO)	2	2	2	2	1	9
School admin./exp. (SCH)	2	2	2	2	0	8
**Total**	**5**	**6**	**5**	**7**	**3**	**26**

admin., administrative staff such as principals, vice principals, and supervisors; exp., experts in special needs and inclusive education.

Across the four study sites, 26 semi-structured interviews – with nine participants from GOs and CSOs each, and eight from schools – were conducted. Inclusive education experts and coordinators at the regional education bureau that governs K-12 education across the region, including the study sites, were also interviewed. Specific criteria to select organisations were used. Among the CSOs, those that operate in at least one of the study sites and have education or student-focused programmes, including Light for Children, Save the Children, Cheshire Foundation, Mahibere Hiwot for Social Development, Yenege Tesfa, World Vision and the UoG-CBR programme were included. For GOs, those that focus on or support school inclusion and accessibility at various levels, including the Women and Children Affairs, the Labour and Social Affairs, and Region, Zone and *Woreda* Education Bureau or Offices were recruited. For schools, a range of schools at different levels of inclusivity and accessibility – in terms of, for instance, having an inclusion resource centre – were targeted.

Finally, specific participants were recruited as key informants based on their position, expertise and knowledge of programmes, collaboration, and support for inclusion in general and inclusive education in particular. The list of participants included SNIE experts, directors, officers, managers and chairpersons in GOs, CSOs and schools.

### Data collection

Before data collection, research ethics boards at Canadian and Ethiopian universities reviewed and approved our study proposal. Researchers adhered to all study procedures and scientific and moral standards for the ethical conduct of research with human participants. Study participants provided verbal consent to their participation in the interviews and audio recordings of the sessions and for the researchers to use the information they provided in aggregated analysis and reporting.

Researchers used semi-structured interview guides to collect qualitative data from the selected individuals in GOs, CSOs and schools (SCH). They developed and finalised the guides in English first, and, native speakers translated them into Amharic. They conducted all interviews in Amharic, the administrative working language and peoples’ lingua franca in the study sites. They briefed all interviewees about the study’s objective and scope, the length of the interview and their rights for confidentiality and withdrawal. For later verbatim transcription, we audio-recorded all interview sessions. Research assistants transcribed the audio recordings of interviews in Amharic. Native Amharic speakers and professional English language teachers at a university in northern Ethiopia translated the Amharic transcripts into English texts.

### Data analysis and presentation

This study employed a qualitative descriptive approach (Sandelowski 2000) to analyse empirical data regarding multistakeholder collaboration to support inclusive education in the four study sites. The researchers employed multiple phases of exploring and organising the empirical data. They started by uploading five English interview transcripts into ATLAS.ti (9) and conducting initial coding independently. They then collectively identified common and unique threads in the initial codes independently created and built a codebook that included all codes. The compiled codebook became the template for coding the remaining 21 interview transcripts. They iteratively created or modified the codebook as they finalised the coding process.

Then, the researchers grouped the codes and their associated quotations into categories and the categories into themes based on their conceptual and empirical relatedness. Given the limited number of CBOs, OPDs and NGOs operating in the study sites, they were merged under a new category that is ‘CSOs’, allowing for a concise presentation and representation of their narratives in the report.

Researchers used participants’ narratives and quotations to describe and contextualise emerging themes on multistakeholder collaboration in inclusive education. They used a unique participant code for each interviewee, containing information on their order (P01, P02, etc.), sector (GO, CSO and SCH) and study site (Chilga, Delgi, Gondar, Qola-Diba and Amhara region). For instance, (P02-CSO, Gondar) refers to the second study participant (P02) recruited from a CSO operating in Gondar city.

### Ethical considerations

Ethical clearance to conduct this study was obtained from the University of Gondar, Institutional Ethical Review Board (No. VP/RTT/05/395/2021).

## Results

We identified five core areas of stakeholders’ collaboration to support the inclusion of students with disabilities in education: (1) school or community accessibility; (2) student enrolment and retention; (3) financial, material and medical support; (4) teacher and student capacity-building; and (5) institutional accountability.

### School or community accessibility

Educational inclusion is only possible when students can physically access school. Nevertheless, a participant reported how, in Gondar, ‘most institutions have buildings constructed before the Proclamation [*requiring accessibility considerations*] was adopted’, requiring collaboration among city administration to improve accessibility. The participant added:

‘We build ramps for some classrooms at those [*school*] support centers … even if some of the ramps have crumbled … We have also made modifications to special needs classrooms … We will make sure that new construction will include accessibility considerations from the very beginning.’ (P33-GO, Gondar)

Another participant from the Regional Education Bureau discussed collaborating with schools and investing in ‘ramps for the newly established schools, making toilets accessible and comfortable to the physically disabled … students, and expanding these practices every year’ (P63-GO, Bahir Dar). Likewise, building community facilities such as roads and other transportation systems for students with disabilities to travel to and from school was also a priority among participants from GOs. For instance, a participant in Chilga highlighted the collaboration between the city administration and the municipality to ensure the accessibility of public spaces:

‘If the ditch is built and opened to the public without considering people with disabilities, an individual who walks with his legs and hands can’t cross the ditch. We facilitated the [*accessible*] crossing to be filed [*with the government*]. Similarly, we initiated a walkway to be built so that students with visual impairments can walk over it using a cane.’ (P13-GO, Chilga)

### Student enrolment and retention

Given low school enrolment, participants talked at length about multiple collaborative efforts to recruit children with disabilities to attend school, including community outreach programmes such as visiting, phoning or incentivising families of children with disabilities. For example, a participant from an NGO described attempts to ‘convince’ families through ‘community gatherings, festivals, and the like’ to send their children with disabilities to school (P06-CSO, Chilga). The participant also talked about improving awareness of ‘inclusive education … at school, home, mosques and churches using both traditional and modern associations’ (P06-CSO, Chilga). Similarly, a civil servant in Chilga shared:

‘We used influential religious fathers to speak about children with disabilities to their congregations … We encourage children to enroll in school through our *Qetena* [*the smallest administrative body in towns and cities*] and village leadership …’ (P13-GO, Chilga)

A civil servant in Delgi concurred about the positive impact similar activities had on the school enrolment of children with disabilities as a result of community awareness efforts, ‘many parents started sending their children with disabilities to public places and schools.’ (P16-GO, Delgi).

Other participants shared stories of efforts to convince families to send their children to school targeted through phone calls. An educator in Qola-Diba, for instance, described a ‘plan to make a call campaign to [*families*] and to make [*students with disabilities*] register for school rather than hiding them’. The participant was among others who also emphasised building relationships with families by collaborating with community programmes such as:

‘[*C*]ommunity-based support and care coalition (the 3Cs) or mechanisms intended to support orphans, the elderly, people with disabilities and human immunodeficiency virus (HIV) positive women [*with special attention to children with disabilities and make sure their support are prioritized before others*] in their neighbourhoods – to facilitate school enrolment.’ (P57-SCH, Qola-Diba)

On the other hand, participants described ‘identify[*ing*] households with children with disabilities’ (P19-CSO, Qola-Diba) through ‘door-to-door mobilization’ (P02-CSO, Chilga). In particular, several participants, including a civil servant in Chilga, described the importance of ‘using individuals who know the neighbourhoods well to explore the number of first-time children with disabilities enrolling in schools the following year’ and accordingly making adequate preparations to accommodate and support them (P13-GO, Chilga). Likewise, an expert from an NGO operating in Chilga indicated deploying:

‘[*T*]wo volunteers’ in collaboration with UoG-CBR’s field workers to re-identify and register students with disabilities that the volunteers identified [*following security-induced school closure*] … We were able to recruit them from every *Kebele.*’ (P07-CSO, Chilga).

In the same vein, a civil servant in Delgi described using official government channels and community social workers to gather information about children with disabilities and promote their school enrolment:

‘When we receive information that children are not willing to go to school or their parents refuse to let them go, we speak with their parents and school teachers and get the children to school.’ (P20-GO, Delgi)

Furthermore, several participants emphasised raising families’ expectations for children with disabilities to encourage school enrolment. For instance, a participant from an NGO engaged in door-to-door outreach programme described using:

‘[*A*] person with a disability, a UoG graduate, as an advocacy officer … to serve as a role model for children with disabilities and … teach them about disability rights.’ (P07-CSO, Chilga)

According to the participant:

‘We were able to register 170 students [*for school*] at first, but more children with disabilities signed up after a month or two by taking him as a role model. Thus, [*the school’s*] number[*s*] reached [*were*] 340.’ (P07-CSO, Chilga)

In addition, a civil servant in Delgi noted:

‘We use every opportunity to tell them about people with disabilities who are now in top [*government*] positions because of their hard work in their studies. We tell them that they have bright minds.’ (P20-GO, Delgi)

Finally, several participants reported efforts to retain students with disabilities in schools once enrolled. For instance, a civil servant in Delgi described efforts to address student attrition by:

‘Asking teachers to tell us the students’ [*with disabilities*] reasons they drop out … If they [*teachers*] identify a problem for [*students*] dropping out of school, we go and consult with their families. We even collaborate with the UoG-CBR program to go to their home and discuss ways to help them.’ (P20-GO, Delgi)

Further, a civil servant in Chilga reported how they use ‘parent-teacher meetings’ to encourage families to keep their children in school:

‘When parents come to visit their children at school, there is a classroom for children with disabilities [*an inclusive education resource centre*] … Parents see their children with disabilities playing with peers. This also helps the parents to believe that their children can fit into different situations.’ (P13-GO, Chilga)

### Financial, material and medical support

Participants reported how organisations collaborated ‘to make sure that children with disabilities receive needed learning materials, medical treatment, and assistive devices …’ (P19-CSO, Qola-Diba) to enhance inclusive education and ‘make sure that the students don’t drop out’ of school. (P03-CSO, Chilga)

#### Financial support

Participants described the importance of ‘pocket money’ (an informal phrase referred to as a monthly stipend the government provides for children with disabilities while in school) in encouraging school enrolment and retention among children with disabilities:

‘Even if it may be a small amount, students come to school if they know only those who come to school regularly will get the money. And if they come to school regularly, they will keep on performing well.’ (P26-GO, AMH)

According to an educator in Delgi, the financial assistance, however, uses flat rates for disbursement by one’s disability type, thereby not meeting the dynamic needs of students with multiple disabilities who may need more support than others (e.g. students ‘who use wheelchairs and who cannot manage their saliva by themselves’ versus ‘those who have hearing impairments’) (P23-SCH, Delgi). In addition, students with disabilities receive governmental financial support only if they attend schools – they go without it if they drop out of school or during the 2-month school closure between academic years. This rule works against many students with an intellectual disability, as a civil servant in Gondar noted:

‘The 2002 EC [*Ethiopian Calendar*] Regional Education Bureau guidelines state that students with intellectual impairments should return to their families if they don’t improve within three years … But the thing is, change or improvement is measured in terms of academic achievement. However, there are many parameters for measuring change … [*including*] their social interactions, communication skills [*and*] physical well-being … However, the guidelines do not allow them to stay in school for more than three years unless they are academically fit.’ (P33-GO, Gondar)

Moreover, students’ monthly stipend and annual allowance do not adequately account for the high inflation and rising cost of living, leaving families with low incomes in dire conditions – especially for rural families who pay for transportation, rent and meals to send their children to accessible urban schools. As a result, many participants, including an NGO representative, reiterated that they ‘work[*ed*] in collaboration’ to ensure that students with disabilities ‘receive the [*pocket*] money on time … make sure that they are paid’ and advocate for ‘what could be done if a person stays away from school for some time’ (P07-CSO, Chilga). For instance, the Ethiopian Centre for Disability and Development, a local NGO, collaborated with government agencies to provide loans to families of children with disabilities to ‘generate income’ for their families. Other participants proudly recounted positive outcomes of their advocacy, such as students enrolling in school and finding ways to more easily access pocket money by ‘open[*ing*] a saving account so that their payment can be credited to it’ and gaining assurances from the government that ‘the [*monthly*] payment shall not be terminated even if a student drops out of school’ (P03-CSO, Chilga).

#### Material support

Participants described collaborative efforts they used to assess the needs of students with disabilities and plan for materials provision. For example, educators and civil servants collected, organised and furnished NGOs with relevant data on students and schools to facilitate support. For instance, a civil servant in Chilga stated:

‘[*NGOs*] don’t just come in and help. They want information. Thus, we first collect the information in each zone. After that, we coordinate the *Kebeles* and evaluate the information together. In addition, in my and the school principals’ presence, we talked about which student with disabilities has critical problems and need urgent support and which don’t.’ (P14-GO, Chilga)

Based on the identified needs, participants reported that educational stakeholders (e.g. Missionaries of Charity and Organisation for Social and Health Development) collaborated to provide students with disabilities with educational materials (e.g. books and pens, Internet access, Braille materials, uniforms, school bags, exercise books, canes, hearing aids and wheelchairs). Civil society organisations also furnished special education ‘resource centres’ with ‘desktop computers with different types of software, like JAWS’. For example, a civil servant in Chilga indicated:

‘[*In collaboration*], we budget for yearly supplies based on the number of children we have in the year … In addition, when donors come … we connect them with concerned children through schools.’ (P13-GO, Chilga)

Other examples of collaborating and advocating for materials included ‘reach[*ing*] out to the *Woreda* administration and asking them to be more inclusive and proactive’ and referring schools to ‘bodies that can cover their [*inclusive*] materials shortage’ (P19-CSO, Qola-Diba). In Chilga, a participant stated:

‘We go to offices such as the education office looking for exercise books and uniforms for the students [*with disabilities*]. They give us materials they obtained from other projects. When there is no uniform, we write a letter to schools so that students can attend their education without uniforms. Still, we buy them uniforms when we get the money …’ (P02-CSO, Chilga)

#### Medical support

Some participants made efforts to provide medical care and materials for personal hygiene and sanitation (diapers, sanitary materials and soaps) to students with disabilities to encourage school participation and performance. For example, a participant discussed how NGOs, UoG and the CBR programme have collaborated to ‘facilitate children [*with disabilities*] to be able to get medical treatment’ at the UoG referral or comprehensive hospitals, or ‘get children with disabilities [*with advanced or unique health problems*] referred to healthcare institutions in Addis Ababa for better treatment’ (P19-CSO, Qola-Diba). Likewise, a civil servant in Delgi described how the Women and Social Affairs Office engaged with health centres and schools to identify students with disabilities requiring medical care and ‘find ways to provide treatment and medication’ (P20-GO, Delgi).

### Teacher and student capacity-building

Participants, including a civil servant, described collaborative efforts to integrate inclusive, disability-related content into training programmes with curricula that otherwise ‘focus on the transfer of theoretical knowledge - disregarding special needs education’ (P63-GO, Amhara). The participant elaborated on how education offices, educational institutions and CSOs collaborated in various *Woredas* of the region to enhance teachers’ capabilities to: (1) assess and determine the level of students’ disabilities; (2) understand how to assist students using assistive technology or provide accommodations (e.g. ‘writing in bold or with a larger font size’, ‘tell them to take the front seats in classes’); and (3) engage in non-biased service delivery and student assessments (P63-GO, Amhara).

Several school staff trained in SNIE and civil servants described experience-sharing and knowledge-sharing among school teachers. For instance, one SNIE participant described a collaboration among the *Woreda* Education Office, UoG-CBR programme and schools to provide ‘sign language training for teachers … [*about*] how a teacher can treat a student with special needs [*due to*] a hearing impairment’ (P33-GO, Gondar). Another SNIE expert insisted fellow teachers treat students with disabilities ‘equally with regular students in the teaching-learning process … in a fatherly and motherly manner’ (P05-SCH, Chilga). Further, a school administrator described developing school-wide inclusive structures based on university and regional inclusive education training and integrating lessons learned from ‘visit[*ing*] exemplary schools like [*the one in*] Hamusit [*a town in South Gondar*]’ (P10-SCH, Chilga). Similarly, a civil servant described training platforms and ‘experience-sharing’ activities to facilitate:

‘[*P*]eer discussions in schools … [*to*] connect children with disabilities with others … [*and*] eliminate the possibility of children with disabilities being ostracized by their schoolmates or in the community outside school.’ (P40-GO, Gondar)

Moreover, participants spoke about collaborations involving universities, NGOs and the government to create inclusive education coursework for teachers’ training programmes. For example, an interviewee stated:

‘It was started at Kotebe Teachers College, Addis Ababa. Then it was incorporated into the curriculum and implemented in Addis Ababa, Dessie, and Gondar Teachers College … It now runs under the name of Moral Education, and many students are recruited to join this program … This course on special needs is offered as a common course in two colleges.’ (P03-CSO, Chilga)

Several participants also described collaborative efforts to build vocational and daily living skills among students with disabilities. For instance, a civil servant described how a GO collaborated with schools to develop ‘life skills training provided for students with intellectual impairment so that they can take care of themselves and develop their communication and social interaction skills’ (P33-GO, Gondar). Other examples of enhancing student vocational skills included training students in ‘handicraft work’ (P33-GO, Gondar) and ‘dyeing, weaving, painting, and sewing’ (P02-CSO, Chilga). Another participant described efforts to support student entrepreneurship and employment:

‘After they [*students with disabilities*] are trained, they return to the *Woreda’s* small business enterprise for a piece of land for work. The *Woreda’s* administrator wrote a letter to them mentioning that each *Kebele* should organize and give a house, land, and workplace for disabled individuals with training.’ (P07-CSO, Chilga)

### Institutional accountability

Participants collaborated ‘to ensure the implementation of inclusive education programs … from the education office down to the school level’ (P40-GO, Gondar). For example, an OPD chairperson underlined their ‘right’ and ‘responsibility to follow up all these [*inclusive education*] endeavors’ to ensure ‘the institutions have implemented what they have included in their [*plans and*] reports’ (P50-CSO, Qola-Diba). Other accountability measures included: (1) ‘check[*ing*] the number of students [*with disabilities*] enrolled in the schools [*and*] the extent to which they are benefiting’; (2) examining ‘the opportunities that the schools in the *Woreda* created for the students to pursue their education’; (3) ‘evaluate[*ing*] the competence of the special needs education teachers and the materials needed for the learning of special needs students’, (P31-CSO, Gondar); and (4) monitoring ‘the provision of learning materials and proper use of the budget to implement inclusive education’ (P50-CSO, Qola-Diba). The chairperson of an OPD described holding ‘education offices and schools responsible when they fail to use the budget accordingly’ and that they ‘challenge them [*education offices*] when they don’t do what they are supposed to’ (P50-CSO, Qola-Diba). An NGO participant also noted how they ‘pressured’ and ‘questioned’ the government ‘why they don’t implement [*relevant regulations*] and hire competent [*special needs and inclusive education*] teachers’ (P31-CSO, Gondar).

Nonetheless, the persistent and ongoing conflict and unrest in the Amhara region affected all the study sites (but more gravely and frequently in Chilga because of the added dimension of ethnicity-based disputes). Participants indicated that violent and widespread conflicts resulted in frequent school closures and student dropouts:

‘Conflicts often occur spontaneously … There were situations when students with disabilities, especially students with mobility problems … were forced to quit [*school*]. Because of those conflicts, they have no guaranteed safety. When a conflict breaks out, one has to run for his or her life. A person with a problem with his [*or*] her legs would be afraid of such situations.’ (P14-GO, Chilga)

Another participant added:

‘There was no peace … This affected children with disabilities [*more*]. Because their parents … do not access information about war or conflict. Children with hearing impairments do not get current information about the war. Children with visual impairments cannot return from school quickly when they receive information about impending conflict … So, [*when schools reopen following conflict*] children with disabilities may be absent from schools for a long time.’ (P13-GO, Chilga)

The cycles of violence also led to reduced inclusive education accountability for promoting inclusive education and supporting students with disabilities. A CSO participant, for instance, lamented how ‘people did not have time to meet and discuss the problems these children faced’ because of the frequent wars and conflicts (P06-CSO, Chilga). Further, the conflict resulted in frequent ‘leadership’ changes, reducing educational collaborators’ expectations and accountability (P06-CSO, Chilga). Because of the level of devastation caused by the conflict, the priority in Chilga and its surroundings became reestablishing order and security and providing relief assistance for the needy whereby the administration started persuading all state and non-state actors to devote resources and efforts to these programmes, forcing ‘some NGOs to end their terms and phased out their projects’ and creating a gap in the support students with disabilities used to receive (P06-CSO, Chilga).

The participant continued:

‘The disabled were among those who suffered the most from internal conflict and instability … The government has allocated a financial budget for disabled students … Nevertheless, since they are not going to school because of the lack of security and peace in the area, their pocket money becomes suspended. No one inquires where, why, and when they have gone.’ (P06-CSO, Chilga)

Given the needed shift in GO and NGO efforts to community relief and security efforts, participants regretfully indicated that the conflict undermined the inclusive education achievements made over the years.

## Discussion

This study explored collaboration among educational stakeholders to make schools inclusive and accessible for students with disabilities in Central Gondar Zone, Ethiopia. It found significant and intersecting educational barriers that students with disabilities experience in Ethiopia, corroborating reports in other studies (Aldersey et al. 2020; Geleta 2019; Jones et al. 2021). For example, although institutions and individuals supported students with disabilities, there were notable gaps and irregularities in promoting inclusive education across study sites. Consistent with existing research, barriers such as inadequate inclusive education resources, SNIE-trained teachers, funding and professional accountability continue to thwart inclusive education (Alemu et al. 2022; Beyene et al. 2020; Tefera et al. 2015; Tiruneh et al. 2019). Participants’ experiences on multistakeholder collaboration also align with prevailing scholarly views on the indispensability of collaboration in removing barriers to inclusive education and supporting for students with disabilities in low-resourced countries such as Ethiopia (Hankebo 2018; Negash & Gasa 2022; Temesgen 2018). For example, as reported in other studies (Franck & Joshi 2017; Gedfie & Negassa 2019; Šiška et al. 2020), participants highlighted the potential of schools leveraging resource centres as focal institutions to promote inclusive education and support students with disabilities. For instance, resource centres could coordinate research-informed capacity-building programmes for teachers and students with disabilities, including creating a cadre of SNIE experts, experience-sharing and cascading SNIE training for all teachers.

On the other hand, participants identified collaborative strategies GOs, CSOs and schools used to increase school enrolment and retention among children with disabilities, including awareness campaigns, home-to-home visits, role modelling, self-advocacy, capacity-building and community campaigns. Participants claimed significant outcomes as a result of these collaborative efforts, as also reported in other studies (Brittanny & Joshi 2017; Hankebo 2018; Tonegawa 2019). Though studies reported the significance of the government’s financial support to students with disabilities when enrolled in school (e.g. Admas 2009; Franck & Joshi 2017), this study identified instances whereby educational collaborators advocating for and extending the financial support into the off-school summer season to encourage school enrolment and retention. Study findings also revealed efforts to support students who exit school through the development of vocational and life skills to reduce their dependence on families, communities or the government.

Finally, during this study, the Amhara region, especially Chilga, was deep into a multifaceted and protracted conflict. Participants spoke of the rippling and deep impacts of persistent instability, which affected school activities as well as collaboration in support of inclusive education and students with disabilities in schools. During this time, conflict surges resulted in spontaneous school closures variable in duration and parents were often uninformed when schools reopened or were reluctant to send their children to school, causing a higher dropout rate among students with disabilities during the conflict period. Furthermore, as NGOs and GOs focus on emergency and rehabilitation programmes, their usual support to schools or students with disabilities waned, dampening the already limited support for inclusive education. However, participants also recognised that government offices, with their significant limitations, continued to support schools in creating community awareness of disability, prompting parents to start sending their children to school after the conflict.

Study findings also provide an opportunity for a tentative systemic analysis of multistakeholder collaboration for inclusive education in improving accessibility, enrolment and retention, support, capacity-building and accountability. Taking schools as systems, three discernible multistakeholder collaboration patterns emerge that initiate or promote inclusive education and services for children with disabilities: structured, semi-structured and unstructured collaboration. Examining study findings in this way will help us understand the modal collaboration platforms that have been functional, or even effective, in promoting inclusive education in the study sites.

Structured collaboration involves sustained, well-organised and monitored alliances or engagements among stakeholders. An example of structured collaboration includes the World Bank-sponsored ‘Ethiopian General Education Quality Improvement Program for Equity’ (E-GEQIP), channelling resources through the Ethiopian government to support and strengthen general education. It uses a signed collaboration agreement with terms outlining party roles in the management, eligibility determination, provision of education support and services, school accessibility and supporting disadvantaged students. Structured collaborations clearly outline eligibility requirements and enable students with disabilities to receive formal support via a system based on transparency, accountability and sustainability. Moreover, such programmes significantly impact the status of inclusive education and the support children with disabilities receive. In other words, schools are more likely to make positive moves towards inclusion when they receive clear goals, structures, programmes, services, expectations, accountability and timelines from their significant environment and collaborators.

Semi-structured collaboration involves minimal management coordination among participants and minimal acknowledgement from a formal agency. This lack of formal oversight can undermine effectiveness, community ownership and sustainability. However, there is typically minimal negotiation or communication with schools to determine eligibility criteria. For instance, UoG, through its CBR and faculty-initiated programmes, often uses a semi-structured format to support and train students with disabilities and to help schools and staff implement inclusive education. Faculty-initiated programmes depend largely on university funding and seldom involve formal agreements with schools or beneficiaries, although there may be some consultation during the initial stages of project planning. In sum, semi-structured collaborations usually lack formalisation and clear expectations, roles and accountability, relying instead on providers’ willingness and resource availability. When visible support declines, these collaborations suffer, ultimately impacting inclusive education and children with disabilities. Nevertheless, semi-structured collaborations are the primary means through which schools provide essential support and services for inclusive education and students with disabilities.

The third type of collaboration, unstructured collaboration, involves organisations or individuals providing services and support to children with disabilities or schools through mechanisms that lack accountability, regularity, formality or sustainability. Unstructured collaboration is often seen in rural schools and typically involves one-time material support without formal negotiations, shared management or ongoing administration (Alemu et al. 2022). This type of collaboration does not fully fit the definition of collaboration, as the providers usually do not engage with schools, educational stakeholders or beneficiaries in planning or delivering these services and support. However, they mobilise resources or adjust their budget to provide assistive devices or educational materials from their sense of civic responsibility and appreciation of inclusive education. Despite being irregular, informal and sporadic, these parties assist schools in filling essential gaps in inclusive education programmes.

Participant data indicate that structured (e.g. monthly stipend and annual allowance students with disabilities receive from the government under eligibility requirements) and semi-structured collaborations (e.g. collaborating on annual community campaigns to recruit students with disabilities to attend school) are the lynchpin of inclusive education and consistent support for children with disabilities in Central Gondar Zone. This finding contrasts with other studies that claimed unstructured services and support are the foundation of inclusive education in Ethiopia (Hankebo 2018; Mergia 2020; Temesgen 2018). A key contributing factor to the relevance and effectiveness of structured or semi-structured collaborations is their verticality, involving parties with different resources or authority levels. Vertical collaborations are usually government-led and integrated into official work and reporting structures, while horizontal collaborations are less common and more challenging to establish. Horizontal collaborations involve parties with commensurate powers or roles in programming and delivery, requiring power negotiations and agenda formulations for fruitful and sustained engagement. We did not find many illustrative instances of such collaboration, indicating the enormity of work remaining to create a consensual and shared agenda among parties towards promoting inclusive education.

## Limitations

In this study, we conducted a qualitative-descriptive analysis of multistakeholder collaborations to support inclusive education in central Gondar, Ethiopia. Our analysis and conclusions relied on data generated via interviews with key informants purposively selected from GOs, CSOs and schools located in Chilga, Delgi, Gondar and Qola-Diba towns. Consequently, our findings and conclusions may not necessarily reflect other stakeholders’ views such as students with disabilities and their parents. Although we also asked participants to provide retrospective and contextual data on inclusive education in their localities, our conclusions reflect the situation during the data collection period. We recommend future researchers explore multistakeholder collaboration through their various development phases of conception to maturity and track how they negotiate their interests, create shared agendas, assume complementary duties, among others, via longitudinal data using multiple research methods and tools to verify and triangulate findings and conclusions.

## Conclusion

In this study, we reported on the key sectors/areas and how educational stakeholders collaborate to promote inclusive education and support students with disabilities using qualitative data collected from key informants in purposively selected GOs, NGOs and schools in the Central Gondar Zone of the Amhara region, Ethiopia. Study results complement existing literature documenting patterns of inclusion and exclusion remain largely unchanged despite significant efforts in policy and programming (Alemu et al. 2022; Beyene et al. 2020; Tefera et al. 2015; Tiruneh et al. 2019). Conversely, we found discernible factors in the Central Gondar Zone that have not yet been extensively explored. For example, we found GOs and NGOs advocating for off-school season financial support for students with disabilities to motivate them and their families to enrol and maintain their children’s attendance. We also uncovered new contributions to the literature, including the negative impact of local instability and conflict on student attendance and inclusive education progression, multistakeholder collaboration, institutional accountability and inclusion support for students with disabilities. In addition, we found greater instances of meaningful multistakeholder structured and semi-structured than unstructured formats. From a systems perspective, schools are more likely to respond to structured or semi-structured support or feedback from their significant environment with formality in expectations, accountability, eligibility requirements and service provisions. Finally, based on our findings and limitations, we recommend further studies on the significance and impact of off-season financial support to students with disabilities and conflict on multistakeholder collaboration, accountability and support for inclusive education.
